# Identification of a novel inflammatory mast cell subpopulation in triple-negative breast cancer

**DOI:** 10.3389/fimmu.2026.1975768

**Published:** 2026-10-01

**Authors:** Zehan Li, Ziqing Wu, Lingxin Zeng, Chuzhong Wei, Huazhen Wu, Huisi Qiu, Jianping Chen, Pingmei Zhang, Yuxiao Tian, Fanghui Bian, Jiemin Liang, Dongqian Li, Muyan Liu, Zhiying Li, Yuyi Liu, Meiyi Li, Xiangfeng Cen, Lihuan Zhou, Huodi Chen, Huijuan Jiang, Haosheng Li, Xin Wang, Ruiming Tang, Song Zhu

**Affiliations:** 1 Department of Oncology, The Affiliated Qingyuan Hospital (Qingyuan People’s Hospital), Guangzhou Medical University, Qingyuan, China; 2 Department of Urology and Institute of Urology and National Clinical Research Center for Geriatrics, West China Hospital, Sichuan University, Chengdu, China; 3 Department of Medical Imaging, The Affiliated Qingyuan Hospital (Qingyuan People’s Hospital), Guangzhou Medical University, Qingyuan, Guangdong, China

**Keywords:** mast cells, NF-κB, single-cell RNA sequencing, triple-negative breast cancer, tumor immunity, tumor microenvironment

## Abstract

**Background:**

Triple-negative breast cancer (TNBC) is associated with poor therapeutic outcomes, and increasing attention has been directed toward the tumor microenvironment (TME). Mast cells are an important component of the TME, yet their heterogeneity and functional roles in TNBC remain incompletely understood.

**Methods:**

Single-cell RNA sequencing data of TNBC were analyzed using the Seurat pipeline. Mast cells were extracted and re-clustered, and the subpopulations were characterized by functional enrichment, transcription factor, pseudotime, drug sensitivity, and cell–cell communication analyses. The role of the inflammatory subpopulation was further examined using an in silico virtual knockout approach and validated in a 4T1 subcutaneous tumor model in BALB/c mice.

**Results:**

Four transcriptionally distinct mast cell subpopulations were identified. Among them, the C4 subpopulation exhibited a pronounced inflammatory signature and was highly enriched in the NF-κB pathway. C4 was predicted to have lower sensitivity to several first-line chemotherapeutic agents and was associated with an unfavorable treatment response. Cell–cell communication analysis suggested that C4 may suppress CD8^+^ T cells through multiple immune-inhibitory signals. *In vivo*, IL-33 administration increased the proportion of C4 and promoted tumor growth, and the proportion of C4 was positively correlated with tumor volume and weight.

**Conclusions:**

We identified an inflammation-associated mast cell subpopulation (C4) that may contribute to TNBC progression, providing a potential target for future therapeutic exploration.

## 1 Introduction

As one of the most frequently diagnosed malignancies in females, breast cancer represents the second leading cause of cancer-related death worldwide, surpassed only by lung cancer (1). Despite the improved five-year survival rates resulting from therapeutic and diagnostic advancements, breast cancer mortality remains substantial due to its escalating incidence and the emergence of drug resistance (2, 3). Underlying these therapeutic challenges is the profound heterogeneity of the disease. To navigate this complexity, breast cancer is clinically stratified based on the expression profiles of the estrogen receptor (ER), progesterone receptor (PR), and human epidermal growth factor receptor 2 (HER2). This receptor-based classification is crucial for guiding personalized therapeutic decisions, thereby significantly enhancing treatment efficacy and improving patient prognoses (4). Notably, triple-negative breast cancer (TNBC)—a subtype devoid of ER, PR, and HER2 expression—represents a distinct clinical challenge. It exhibits highly aggressive biological behaviors, which translate into a worse prognosis, an elevated risk of recurrence, and an earlier onset of relapse when juxtaposed with other molecular subtypes (5–7). In recent years, immunotherapy has emerged as a promising therapeutic strategy for TNBC patients. Compared to other breast cancer subtypes, TNBC is characterized by a higher degree of T-cell infiltration, rendering it more responsive to immunotherapeutic interventions and allowing patients to derive greater clinical benefit (8, 9). Consequently, the widespread clinical application of immunotherapy has increasingly directed research focus toward the tumor microenvironment (TME), given its profound correlation with therapeutic efficacy.

As pivotal orchestrators within the TNBC microenvironment, mast cells serve as essential mediators that profoundly regulate localized immune responses and dictate tumor growth (10–12). Nevertheless, the function of mast cells within the tumor milieu is a subject of ongoing debate. Serving as distinct regulators in the TME, they demonstrate a paradoxical nature, capable of orchestrating both tumor-promoting and tumor-suppressing processes. On the one hand, mast cells exert anti-tumorigenic effects by stimulating immune responses, recruiting cytotoxic T cell, and enhancing immune surveillance via tumor antigen presentation. On the other hand, they can act in a pro-tumorigenic capacity by releasing mediator that induce the recruitment of other immune cells to the tumor microenvironment and stimulate epithelial-mesenchymal transition (EMT) (13–15).

Driven by technological leaps, notably the widespread application of single-cell transcriptomics, emerging evidence suggests that the dual functionality of mast cells may stems from their heterogeneity across various subpopulations (16, 17). In a notable study, Wu et al. characterized an antigen-presenting mast cell cluster via single-cell RNA sequencing (scRNA-seq), confirming its anti-tumorigenic properties achieved through antigen presentation and the subsequent activation of T lymphocytes (18). Herein, we extend the current understanding of mast cell heterogeneity. By integrating single-cell profiling with rigorous *in vivo* experiments, we identified a novel inflammatory mast cell subset and confirmed its pro-tumorigenic capacity. We propose that a more comprehensive classification of mast cell subpopulations is imperative; such insights are crucial for deciphering their paradoxical functions and will ultimately provide a critical foundation for developing more effective, targeted immunotherapeutic strategies.

## 2 Materials and methods

### 2.1 Single-cell RNA sequencing data acquisition, processing, and cell type annotation

The scRNA-seq dataset GSE266919 was obtained from the Gene Expression Omnibus (GEO) database (https://www.ncbi.nlm.nih.gov/geo/) (19). This dataset comprises scRNA-seq profiles of 78 tumor biopsy samples collected from 44 patients with advanced TNBC receiving various chemotherapy- and immunotherapy-based regimens, with libraries generated on the 10x Genomics Chromium platform. The human tumor-derived expression matrices, provided as count files separated by major immune lineage, were used for the present study.

All data processing was performed in R using the Seurat package (v5). The lineage-specific expression matrices were imported and combined into a single Seurat object, in which genes detected in fewer than three cells and cells expressing fewer than 200 genes were excluded. Because these lineage-specific matrices were derived from the same scRNA-seq dataset and were generated and processed using a consistent experimental and computational workflow, their merging did not introduce an additional technical batch. Therefore, no additional batch-effect correction was applied. The merged data were normalized using the LogNormalize method with a scale factor of 10,000, and the top 2,000 highly variable genes were identified with the variance-stabilizing transformation (“vst”) method. The data were subsequently scaled, and principal component analysis (PCA) was performed; the first 30 principal components were used for uniform manifold approximation and projection (UMAP) to visualize the cellular landscape in two dimensions.

Cell types were annotated according to the original cell-type annotations provided with the dataset and were consolidated into eight major populations: T cells, B cells, NK cells, monocytes, macrophages, conventional dendritic cells (cDCs), plasmacytoid dendritic cells (pDCs), and mast cells. The reliability of these annotations was confirmed using a heatmap depicting the expression of canonical lineage-specific marker genes, including CD3D, CD3E and CD2 for T cells; CD79A, MS4A1 and MZB1 for B cells; GNLY and NKG7 for NK cells; KIT, CPA3, TPSAB1 and TPSB2 for mast cells; LILRA4, CXCR3 and IRF7 for pDCs; CLEC9A, CD1A and CLEC10A for cDCs; C1QA, C1QB, APOE and TREM2 for macrophages; and FCN1, S100A8, S100A9 and VCAN for monocytes. Mast cells, as defined in the original annotation, were then extracted from the dataset for all subsequent analyses.

### 2.2 Identification and annotation of mast cell subpopulations

To resolve the heterogeneity within the mast cell compartment, the extracted mast cells were subjected to an independent re-analysis. Highly variable genes were re-identified within this subset using the variance-stabilizing transformation (“vst”) method, after which the data were scaled and PCA was performed. Based on the PCA results, the first 15 principal components were retained for dimensionality reduction and clustering; UMAP was applied for two-dimensional visualization, and unsupervised clustering was performed using the FindNeighbors and FindClusters functions in Seurat at a resolution of 1.0.

Mast cell subpopulations were subsequently annotated by combining two complementary strategies. First, the canonical subset-defining marker genes reported by Wu et al. were employed to assign the previously described mast cell subsets, namely the metabolic/translational-related subset (C1), the stress-response subset (C2), and the antigen-presenting mast cell subset (C3, apMC) (18). Second, to characterize subsets beyond those previously reported, cluster-specific differentially expressed genes were identified in an unbiased manner using the FindAllMarkers function (only.pos = TRUE, min.pct = 0.25, logfc.threshold = 0.25), and the top-ranked markers of each cluster were evaluated. Integrating the literature-based markers with the actual clustering structure, four transcriptionally distinct mast cell subpopulations were ultimately delineated—C1 (metabolic/translational), C2 (stress-response), C3 (antigen-presentation), and an inflammatory subset (C4). The identity of each subset was further corroborated by the expression of representative marker genes, including NDUFA1, COX5B and CTSG for C1; ZFP36, BTG2, FOS and JUN for C2; CD74, HLA-DRB1 and STAT1 for C3; and ICAM1, REL and BCL2A1 for C4.

### 2.3 Functional enrichment analysis

To investigate the biological functions associated with each mast cell subpopulation, Gene Ontology (GO) and Kyoto Encyclopedia of Genes and Genomes (KEGG) enrichment analyses were performed for the four subsets (C1–C4). For each subset, cluster-specific marker genes were identified using the FindAllMarkers function in Seurat (only.pos = TRUE, min.pct = 0.1, logfc.threshold = 0.1) with the Wilcoxon rank-sum test, and the top 200 genes ranked by average log2 fold change were selected as the input gene set. Gene symbols were converted into Entrez identifiers using the org.Hs.eg.db annotation database.

GO enrichment analysis of biological processes and KEGG pathway enrichment analysis were conducted using the clusterProfiler package. The Benjamini–Hochberg method was applied for multiple-testing correction, and an adjusted p-value < 0.05 was set as the threshold for statistical significance. The top five enriched terms of each subset were selected and visualized as heatmaps using the ComplexHeatmap package, in which the enrichment significance was represented as the −log10-transformed adjusted p-value.

### 2.4 *In silico* virtual knockout analysis

To explore the downstream transcriptional impact of key genes at single-cell resolution, an in silico virtual knockout analysis was performed using the scTenifoldKnk package. For each target gene, single-cell gene regulatory networks were constructed from the raw count matrix, with the gene set restricted to the top 3,000 highly variable genes together with the target gene; the silencing of the target gene was then simulated computationally, and the perturbed transcriptional state was compared with the corresponding reference state to estimate the gene expression changes induced by the loss of the target gene.

Genes associated with the perturbation were identified and ranked according to their predicted perturbation response. The most strongly altered genes were visualized using bar plots, and the global differential regulation was depicted using volcano plots, in which genes with an absolute Z-score > 2 and an adjusted p-value < 0.05 were defined as significantly altered; the proportions of significant and non-significant genes were summarized to evaluate the overall extent of the transcriptional shift induced by the virtual knockout.

### 2.5 Cell–cell communication analysis

To explore the intercellular communication between mast cell subpopulations and other cell types within the TME, cell–cell communication analysis was performed using the CellChat package. The four annotated mast cell subsets (C1–C4) were integrated back into the global Seurat object containing all major cell populations, and any mast cells excluded during the purification step were removed. To ensure adequate statistical power, the remaining cell populations were consolidated into the following groups: CD4+ T cells, CD8+ T cells, B cells, NK cells, DCs, macrophages, and monocytes.

A CellChat object was created from the normalized expression matrix, and the human ligand–receptor interaction database (CellChatDB.human) was used as the reference. Overexpressed signaling genes and ligand–receptor interactions were identified, and the communication probabilities were computed using the truncated mean of gene expression (trim = 0.1) to estimate the ensemble of cellular interactions. Interactions supported by fewer than 20 cells in a given population were filtered out. Communication probabilities were then aggregated at the signaling pathway level, and the overall communication network was computed. The number and strength of interactions were visualized using circle plots and heatmaps. Network centrality was calculated with netAnalysis_computeCentrality. Global communication patterns were identified using the CellChat identifyCommunicationPatterns function, which applies non-negative matrix factorization to pathway-level communication probability matrices. The optimal number of patterns was evaluated using selectK, and two incoming and three outgoing patterns were retained. Incoming patterns represent groups of recipient cells with similar signaling-reception profiles, whereas outgoing patterns represent groups of sender cells with similar signaling-secretion profiles. The contribution of each cell group and signaling pathway to individual patterns was subsequently visualized using heatmaps and river plots.signalingsignalingsignaling.

### 2.6 Transcription factor regulatory network analysis

To characterize the transcriptional regulatory programs underlying the mast cell subpopulations, single-cell regulatory network inference was performed using the SCENIC workflow. The raw count matrix of the annotated mast cells was used as input, and genes expressed at very low levels or in too few cells were removed by the built-in gene-filtering step. Co-expression modules between transcription factors and their potential target genes were inferred with GENIE3 using the log-transformed expression matrix. Candidate modules were subsequently refined into regulons by RcisTarget, which retains only the target genes harboring the corresponding transcription factor binding motif within their promoter regions, based on the human motif annotation database.

The activity of each regulon in every cell was quantified as an area under the curve (AUC) score using AUCell, and regulon activity was further binarized into “on” and “off” states. The mean regulon activity of each mast cell subset was calculated, and subset-associated regulons were selected by integrating the most active, the most variable, and the most subset-specific regulons; the resulting regulon activity profiles were visualized as a heatmap. To identify the core regulators, the number of target genes per transcription factor was quantified, and the top five transcription factors with the largest regulons were defined as hub transcription factors. Their regulatory relationships were depicted as a transcription factor–target gene network, and the activity, binarized activity, and expression of these hub transcription factors were displayed on the UMAP embedding.

### 2.7 Pseudotime trajectory analysis

To reconstruct the potential differentiation trajectory of the mast cell subpopulations, pseudotime analysis was performed using the Monocle 2 package. The raw count matrix of the annotated mast cells, together with the corresponding cell metadata and gene annotations, was used to construct a CellDataSet object, with the expression family specified as negative binomial to accommodate unique molecular identifier–based count data. Size factors and dispersions were estimated, and the genes governing the trajectory were selected by performing a differential gene expression test across the four mast cell subsets (C1–C4); genes with a q-value < 0.01 were retained as ordering genes. Dimensionality reduction was subsequently conducted using the DDRTree algorithm, and the cells were ordered along the inferred trajectory to assign each cell a pseudotime value. The reconstructed trajectory was visualized with cells colored by subset and by pseudotime.

### 2.8 Drug sensitivity analysis of mast cell subpopulations

To evaluate and compare the predicted drug responses of the different mast cell subpopulations, drug sensitivity analysis was performed using the Beyondcell package. The annotated mast cell object was analyzed with the Sensitivity Signatures Collection (SSc) as the drug signature reference, which is derived from the Genomics of Drug Sensitivity in Cancer (GDSC) database and captures the transcriptional differences between drug-sensitive and drug-resistant cell lines. For each cell, a Beyondcell Score (BCS) was computed for every drug signature using the bcScore function (expression threshold = 0.1), generating a cell-by-drug BCS matrix in which a higher score denotes a greater predicted sensitivity to the corresponding compound.

To mitigate technical confounding, the BCS matrix was corrected for the number of detected genes per cell using the bcRegressOut function, and UMAP was subsequently performed on the regressed BCS matrix to group cells with similar predicted drug-response profiles. Drug-ranking statistics were then computed for the four mast cell subsets (C1–C4) using the bcRanks function, which prioritizes compounds according to their switch point and mean residual; the mean residual reflects the predicted drug-response profile of a given subset relative to the remaining subsets, with positive and negative values indicating relatively higher and lower predicted sensitivity, respectively. For each subset, the differentially sensitive and differentially resistant compounds were visualized using four-quadrant plots. In addition, the BCS distribution of selected breast cancer–related agents was compared across the four subsets and displayed as histograms, and the per-cell BCS of representative compounds was projected onto the UMAP embedding.

### 2.9 Cell culture

The 4T1 murine mammary carcinoma cell line was originally obtained from the Cell Bank of the Chinese Academy of Sciences and was kindly provided to our laboratory by Dr. Huisi Qiu. The cell line was authenticated before use and was routinely tested for mycoplasma contamination during culture. Cells were maintained in RPMI-1640 medium containing 10% (v/v) fetal bovine serum (FBS) and penicillin–streptomycin at a final concentration of 100 units/mL. All cultures were kept at 37 °C in a humidified atmosphere with 5% CO_2_.

### 2.10 *In vivo* mouse model

Six-week-old female BALB/c mice were purchased from the Guangdong Medical Laboratory Animal Center. All animals were housed under a 12-h light/dark cycle at an ambient temperature of 20–22 °C and a relative humidity of 60 ± 10%. To establish a subcutaneous tumor model, 2 × 10^5^ 4T1 cells suspended in 100 μL of phosphate-buffered saline (PBS) were implanted subcutaneously into each mouse. The *in vivo* experiments were independently repeated three times, with five mice per group in each experiment. Body weights were comparable between the control and IL-33-treated groups at the beginning of each experiment, with no significant between-group difference (P > 0.05). Each mouse was considered an independent biological replicate. The mice were randomly assigned to the treatment and control groups (n = 5 per group). Seven days after implantation, the mice in the treatment group received intraperitoneal injections of recombinant murine IL-33 (cat. no. 210-33-10, PeproTech) at a dose of 0.2 μg per mouse dissolved in 100 μL of PBS, while the control group received an equal volume of PBS; injections were administered every other day from day 7 to day 15. Once tumors became palpable, the longest (L) and shortest (W) diameters were measured every two days using a caliper, and the tumor volume was calculated according to the formula 0.5 × L × W². The predefined experimental endpoint was three weeks after tumor implantation, or earlier if the maximum tumor diameter reached 20 mm, marked tumor necrosis developed, or the animal exhibited a severe adverse clinical condition. At the experimental endpoint, the mice were euthanized by CO_2_ inhalation in a euthanasia chamber, with CO_2_ introduced at a gradual displacement rate of 30% of the chamber volume per minute, and the tumors were subsequently excised and weighed. All animal experiments were conducted in accordance with a protocol approved by the Experimental Animal Ethics Committee of the Affiliated Qingyuan Hospital of Guangzhou Medical University (approval no. LAEC-2025-079).

### 2.11 Flow cytometry

Tumors from individual mice were processed and analyzed separately without pooling. Each flow-cytometric measurement represented one tumor derived from one individual mouse, with five tumors per group analyzed in each independent experiment. Freshly excised tumor tissues from mice were preserved in tissue storage solution (abs9474, Absin) at 4 °C and processed within 24 h. The tissues were immersed in a digestion solution (abs9482, Absin) and incubated at 37 °C for 1 h on a horizontal shaker to facilitate tissue dissociation, and the resulting suspension was filtered through a 70-μm cell strainer to obtain a single-cell suspension. Erythrocytes were removed by incubation with red blood cell lysis buffer (R1010, Solarbio) for 10 min at room temperature, after which 1 × 10^6^ cells per sample were collected for subsequent analysis.

Cells were first incubated with a BeyoFC FcR blocking reagent (anti-mouse CD16/CD32; C1755S, Beyotime) for 20 min on ice in the dark to block Fc receptors. The following fluorochrome-conjugated anti-mouse antibodies were then added sequentially for surface marker staining: FITC-conjugated anti-CD45 (STARTER), Alexa Fluor 647–conjugated anti-CD117 (STARTER), PE-conjugated anti-FcϵRIα (Thermo Fisher Scientific), and BB700-conjugated anti-ICAM-1 (BD Pharmingen); all antibodies were used at the volumes recommended by the manufacturers, and the cells were incubated for 20 min in the dark. Cells stained with the corresponding isotype-control antibodies served as negative controls. Because all four available fluorescence channels in the experimental panel were occupied by CD45, CD117, FcϵRIα, and ICAM-1, an additional viability dye was not included. Therefore, dead cells were not directly discriminated using a viability marker; instead, cellular debris and events with abnormal forward- and side-scatter characteristics were excluded during gating, followed by exclusion of doublets based on FSC-A/FSC-H. After staining, the cells were washed with PBS and transferred to flow cytometry tubes. Data were acquired on a BD Accuri C6 Plus flow cytometer and analyzed using FlowJo software. For quality control, unstained and single-stained controls were included to assess background fluorescence and fluorescence spillover across channels and to assist in establishing gating boundaries, while appropriate negative controls were used to support the identification of positive populations. The flow cytometer was routinely maintained and performance-checked by trained personnel, and all samples were analyzed using the same sequential gating strategy. CD45^+^ immune cells were first identified, mast cells were subsequently defined as FcϵRIα^+^CD117^+^ cells, and ICAM-1-high mast cells were further identified within the mast-cell population according to ICAM-1 expression.

### 2.12 Statistical analysis

Biological replicates were defined as individual mice. The *in vivo* experiments were independently repeated three times, with five biological replicates per group in each experiment. Tumor samples from different animals were analyzed independently and were not pooled. All statistical analyses and data visualization were performed in R. ScRNA-seq data processing and downstream analyses were conducted using the R packages described above. For comparisons between two groups, the Wilcoxon rank-sum test was applied for non-normally distributed data, and the Student’s t-test was used for normally distributed data. Differentially expressed genes in the scRNA-seq analysis were identified using the Wilcoxon rank-sum test. Correlations between variables were assessed using Pearson’s correlation analysis. For functional enrichment analysis, p-values were adjusted for multiple testing using the Benjamini–Hochberg method. Quantitative experimental data are presented as the mean ± standard error of the mean (SEM). A two-sided p-value < 0.05 was considered statistically significant, and significance levels are denoted as follows: *p < 0.05, **p < 0.01, and ***p < 0.001.

## 3 Results

### 3.1 Single-cell landscape of breast cancer and identification of mast cells

A total of 447,494 cells were obtained from the GSE266919 dataset. After quality control, during which genes detected in fewer than three cells and cells expressing fewer than 200 genes were excluded, the retained cells were annotated and classified into eight major cell populations: T cells, B cells, NK cells, pDCs, cDCs, macrophages, mast cells, and monocytes (**Figure 1A**). Each population showed high expression of its corresponding canonical marker genes, confirming the reliability of the annotation (**Figure 1B**). Mast cells were then extracted for subsequent analyses, yielding 1,757 high-quality mast cells. Following UMAP-based dimensionality reduction, these mast cells were further divided into four transcriptionally distinct subpopulations (**Figure 1C**), and the characteristic marker genes of each subpopulation are shown in **Figure 1D**.

**Figure 1:**
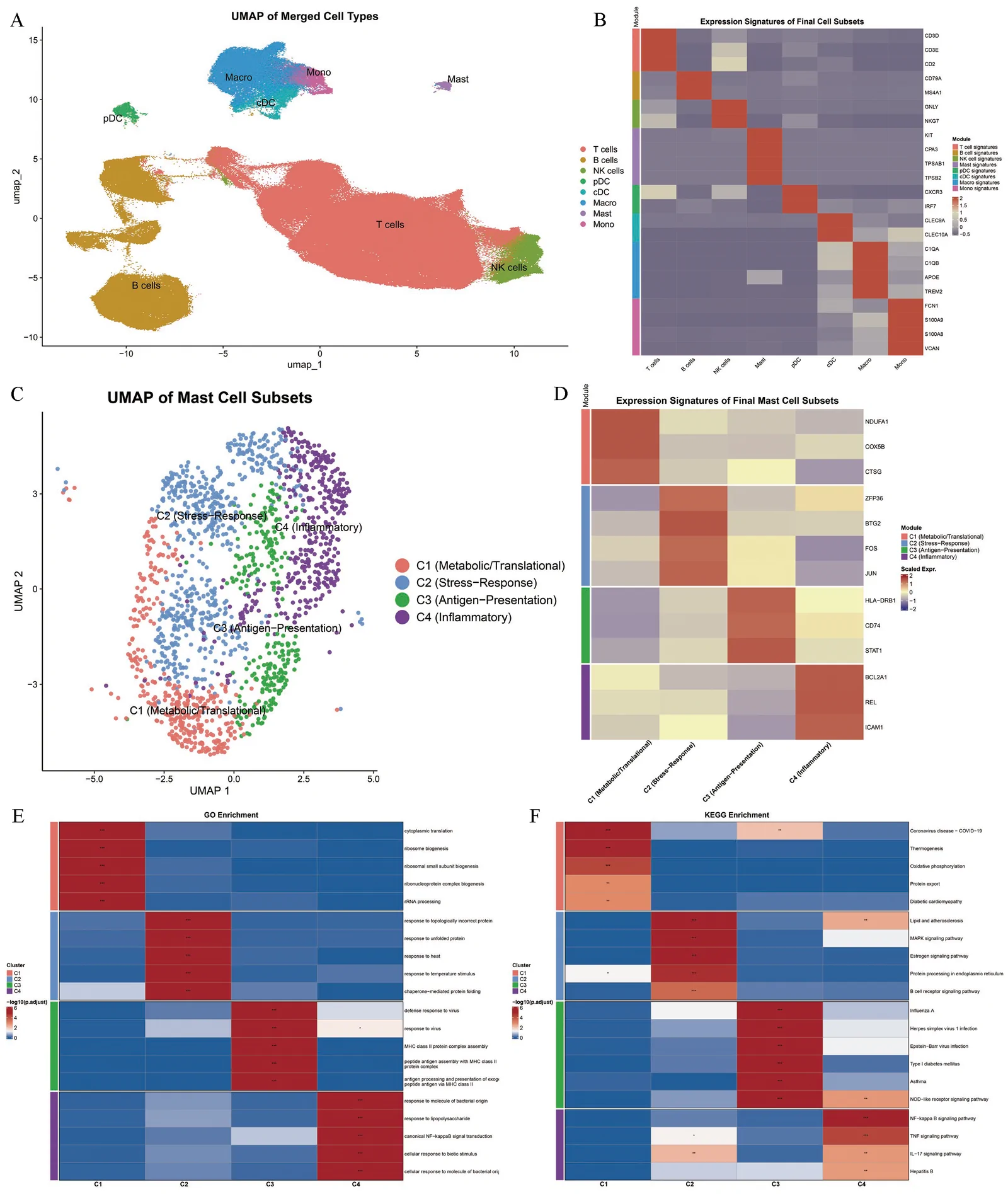
Single-cell landscape of the TNBC tumor microenvironment and identification of four mast cell subpopulations. **(A)** UMAP plot of all cells from the GSE266919 dataset, colored by eight major cell types. **(B)** Heatmap of canonical marker gene expression across the eight major cell types. **(C)** UMAP plot of mast cells, colored by four subpopulations: C1 (Metabolic/Translational), C2 (Stress-Response), C3 (Antigen-Presentation), and C4 (Inflammatory). **(D)** Heatmap of representative marker genes for mast cell subpopulations C1–C4. **(E)** Heatmap of the top five enriched GO biological process terms for each subpopulation. **(F)** Heatmap of the top five enriched KEGG pathways for each subpopulation.

### 3.2 The C4 (Inflammatory) subpopulation is characterized by an inflammatory response signature

The C4 (Inflammatory) subpopulation was characterized by the marker genes ICAM1, REL, and BCL2A1, which have been reported to be associated with inflammatory responses and the NF-κB signaling pathway, suggesting that this subpopulation may be related to inflammation (20–22). Consistent with this, GO and KEGG enrichment analyses revealed that the four mast cell subpopulations were enriched in markedly distinct pathways (**Figures 1E, F**). Notably, C4 was predominantly enriched in NF-κB–centered inflammatory pathways, including the NF-κB signaling pathway, the TNF signaling pathway, and the IL-17 signaling pathway, indicating that the biological function of this subpopulation is dominated by the induction of inflammatory responses (23–25). In addition, C4 was enriched in pathways related to the response to bacterial stimuli, such as “response to molecule of bacterial origin”, “response to lipopolysaccharide”, and “cellular response to biotic stimulus”, suggesting that this subpopulation may be involved in the recognition of and signal transduction in response to inflammatory stimuli.

To further explore the regulatory role of ICAM1, an in silico virtual knockout of ICAM1 was performed (**Supplementary Figures S1A, B**). Among the genes whose regulation was significantly perturbed upon ICAM1 knockout, KDM6B is known to play an important role in inflammation (26), while VIM-AS1、ZEB2、AHNAK、MYH9 and FMNL3 have been reported to be involved in the epithelial–mesenchymal transition (EMT) (27–31). In addition, KIT, a major regulator of the mast cell lineage, was also among the perturbed genes, suggesting a potential link between ICAM1 and the normal function of mast cells (32). Notably, HOOK2, the most strongly perturbed gene, is an adaptor protein involved in microtubule-mediated transport, and its knockdown has been shown to suppress tumor growth and migration in ovarian cancer (33, 34).

### 3.3 Mast cells act as intermediary mediators within the tumor microenvironment

Cell–cell communication analysis of the TNBC tumor microenvironment (TME) revealed extensive interactions between the C4 (Inflammatory) subpopulation and other cell types (**Figures 2A, B**). Among these, DCs, macrophages, and monocytes constituted the major sources of incoming signals to C4 (**Figure 2C**), whereas CD8^+^ T cells were the principal recipients of its outgoing signals (**Figure 2D**).

**Figure 2:**
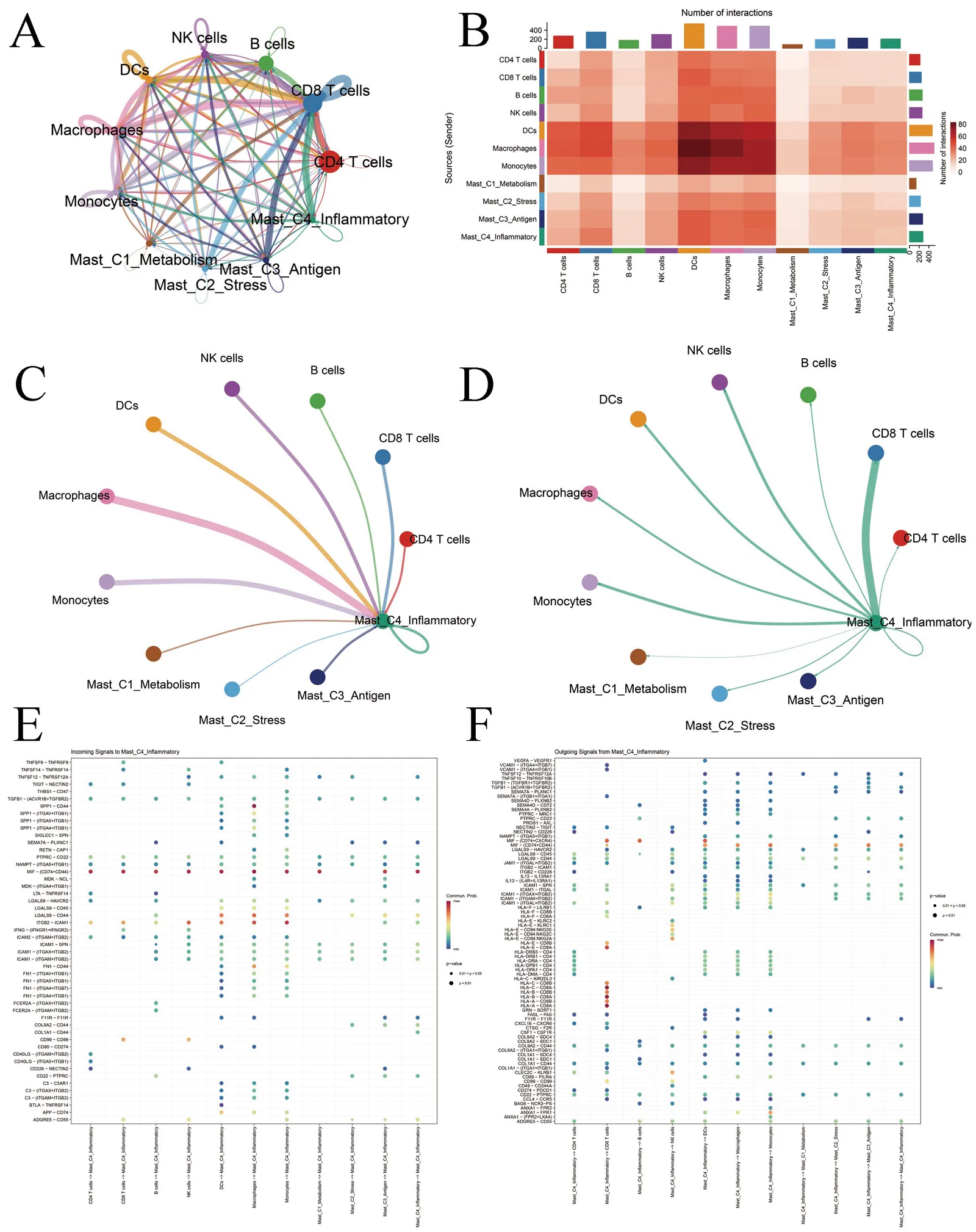
Cell–cell communication between mast cell subpopulations and other cell types in the TNBC tumor microenvironment. **(A)** Circle plot showing the interaction strength among the four mast cell subpopulations (C1–C4) and the other major cell types. Edge width is proportional to the inferred communication strength. **(B)** Heatmap of the number of interactions between cell types, with senders in rows and receivers in columns. **(C)** Circle plot of the incoming signals received by the C4 (Inflammatory) subpopulation from other cell types. **(D)** Circle plot of the outgoing signals sent from the C4 (Inflammatory) subpopulation to other cell types. **(E)** Bubble plot of the significant ligand–receptor pairs mediating incoming signals to the C4 (Inflammatory) subpopulation. Dot color indicates the communication probability and dot size indicates the p-value. **(F)** Bubble plot of the significant ligand–receptor pairs mediating outgoing signals from the C4 (Inflammatory) subpopulation. Dot color indicates the communication probability and dot size indicates the p-value.

Analysis of the incoming and outgoing pathways showed that the incoming signals to C4 were mainly mediated by pathways such as MIF, SPP1, LGALS9, C3, APP, ICAM, and NAMPT, which are involved in immune suppression, immune evasion, EMT and pro-tumor and pro-inflammatory processes (**Figure 2E**) (35–40). Notably, the TNFSF12(TWEAK)–TNFRSF12A(Fn14) pathway is involved in the activation of the NF-κB inflammatory pathway, which is highly consistent with the intrinsic inflammatory function of C4 (41, 42). Regarding the outgoing signals from C4, in addition to the classical MHC class I molecules (mainly HLA-A, HLA-B, and HLA-C), several pathways including CD274, LGALS9, NECTIN2, FASL, and MIF were identified, which are involved in T-cell exhaustion, T-cell suppression, and T-cell killing (**Figure 2F**) (43–47). In addition, the outgoing signals included the ICAM and VCAM pathways. As NF-κB–induced cell adhesion molecules, ICAM-1 and VCAM-1 mediate the tumor cell–endothelial interactions required for extravasation, thereby contributing to metastatic progression (48).

Global communication pattern analysis further identified two major incoming and three outgoing signaling programs. Incoming Pattern 1 was predominantly associated with macrophages, DCs, and monocytes, representing a myeloid-dominant signal-reception program, whereas Incoming Pattern 2 included the four mast-cell subsets together with B, NK, CD4+ T, and CD8+ T cells and was characterized by cell-contact, adhesion, immune-recognition, and immune-regulatory signals (**Figures 3A, B**; **Supplementary Figure S1C**). checkThe major incoming signals received by C4 included galectin, ITGB2, SPP1, FN1, and collagen (**Figure 3C**). In the outgoing analysis, lymphoid cells, myeloid cells, and mast cells were predominantly associated with Patterns 1, 2, and 3, respectively. Notably, all four mast-cell subsets shared Outgoing Pattern 3, suggesting a conserved mast-cell communication backbone despite their distinct transcriptional states (**Figures 3D, E**; **Supplementary Figure S1D**), and the major outgoing signals from C4 included MHC-I, MIF, ICAM, ANNEXIN, and CSF (**Figure 3F**).

**Figure 3:**
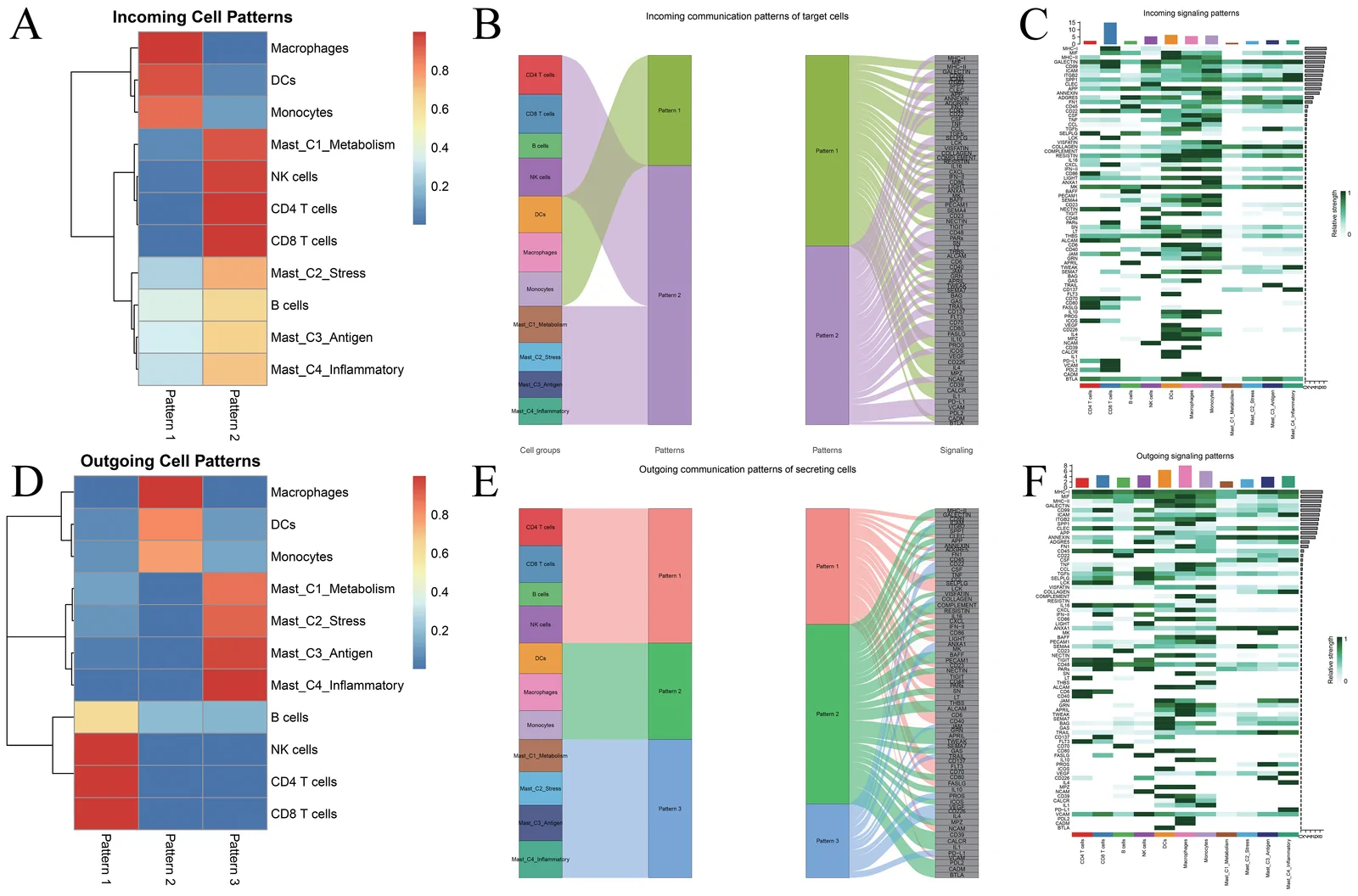
Global incoming and outgoing communication patterns across cell types in the TNBC tumor microenvironment. **(A)** Heatmap showing the contribution of each cell type to the two identified incoming communication patterns. The color scale indicates the relative contribution. **(B)** River plot linking cell groups to incoming communication patterns and the associated signaling pathways. **(C)** Heatmap of incoming signaling patterns, showing the relative strength of each signaling pathway across cell types. **(D)** Heatmap showing the contribution of each cell type to the three identified outgoing communication patterns. The color scale indicates the relative contribution. **(E)** River plot linking cell groups to outgoing communication patterns and the associated signaling pathways. **(F)** Heatmap of outgoing signaling patterns, showing the relative strength of each signaling pathway across cell types.

Through transcription factor analysis, we found that the C4 subpopulation was enriched in transcription factors that constitute the NF-κB family or play important roles in its signal transduction, including NFKB1, NFKB2, REL, BCL3, and NFAT5 (**Figure 4A**), further confirming the distinct role of C4 in the NF-κB signaling pathway (49–51). In addition, C4 was enriched in transcription factors such as ATF3, FOSB, and JUNB, which are key members of the activator protein 1 (AP-1) family; AP-1 is involved in cell cycle regulation, proliferation, and tumorigenesis (52, 53). Finally, we also identified several pro-inflammatory transcription factors enriched in C4, including XBP1, CREM, and BHLHE40 (54–56).

**Figure 4:**
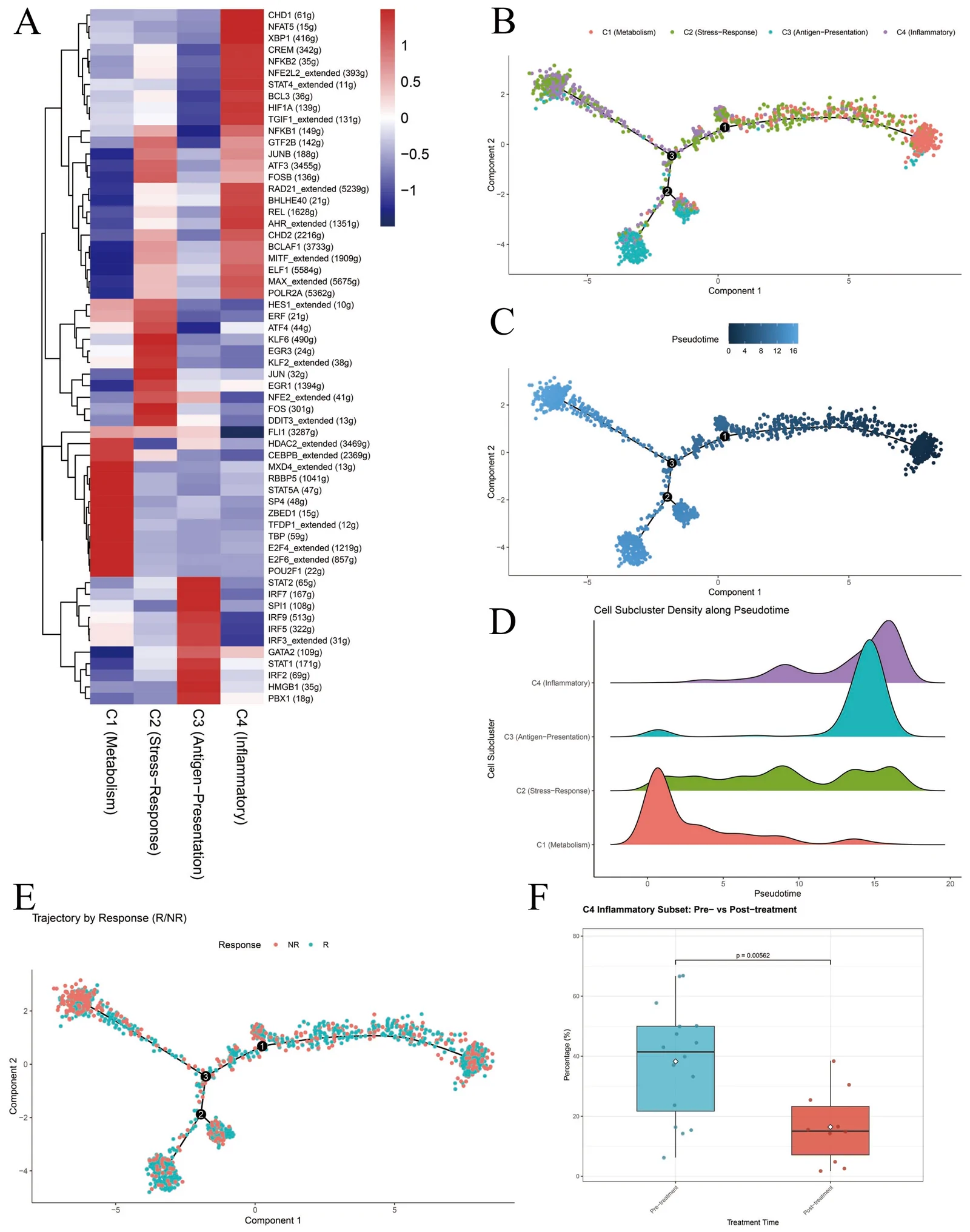
Transcription factor regulatory programs and pseudotime trajectory of mast cell subpopulations. **(A)** Heatmap of the regulon activity across the four mast cell subpopulations (C1–C4), as inferred by SCENIC. The number of predicted target genes is indicated in parentheses after each transcription factor, and the color scale represents the scaled regulon activity. **(B)** Pseudotime trajectory of mast cells reconstructed by Monocle 2, with cells colored by subpopulation. **(C)** The same trajectory as in **(B)**, with cells colored by pseudotime. **(D)** Ridge plot showing the density distribution of each mast cell subpopulation along the pseudotime axis. **(E)** Pseudotime trajectory with cells colored by treatment response (R, responder; NR, non-responder). **(F)** Box plot comparing the proportion of the C4 (Inflammatory) subpopulation between pre-treatment and post-treatment samples.

### 3.4 The C4 (Inflammatory) subpopulation is associated with treatment response

In the pseudotime analysis of the mast cell subpopulations, C1 (Metabolic/Translational) was observed to be positioned at the origin of the trajectory, from which two distinct branches extended, giving rise to the C3 (Antigen-Presentation) and C4 (Inflammatory) subpopulations, respectively (**Figures 4B–D**). After incorporating the clinical information on treatment response, we found that the branch containing C4 was enriched for non-responding patients, whereas the branch containing C3 was enriched for responding patients (**Figure 4E**). Analysis of the proportion of C4 before and after treatment further showed that the proportion of C4 decreased following treatment (**Figure 4F**). After analyzing patients receiving different treatment regimens, we found that, with the exception of the C2 subset, the distributions of the other mast-cell subsets, including C4, did not differ significantly among treatment groups (**Supplementary Figure S1E**), suggesting that this mast-cell subpopulation structure is broadly preserved across different treatment regimens.

In the drug sensitivity analysis, C4 was predicted to have lower sensitivity to vorinostat, elesclomol, sabutoclax, CD-437, and FK-866, whereas it was predicted to have higher sensitivity to VX-11E, dactinomycin, rigosertib, gemcitabine, and JQ-1:MK-0752 (**Figures 5A, B**). Furthermore, analysis of commonly used chemotherapeutic agents revealed that C4 was associated with lower predicted sensitivity to several first-line chemotherapeutic drugs for TNBC, including vinorelbine (**Figures 5C, D**), epirubicin (**Figures 5E, F**), and paclitaxel (**Supplementary Figures S2A, B**), whereas gemcitabine was associated with a higher predicted sensitivity profile in C4 (**Supplementary Figures S2C, D**). Interestingly, analysis of the PARP inhibitor olaparib (**Supplementary Figures S2E, F**) and the PI3K inhibitor alpelisib (**Supplementary Figures S2G, H**) showed that C4 was also predicted to have relatively higher sensitivity to these two agents.

**Figure 5:**
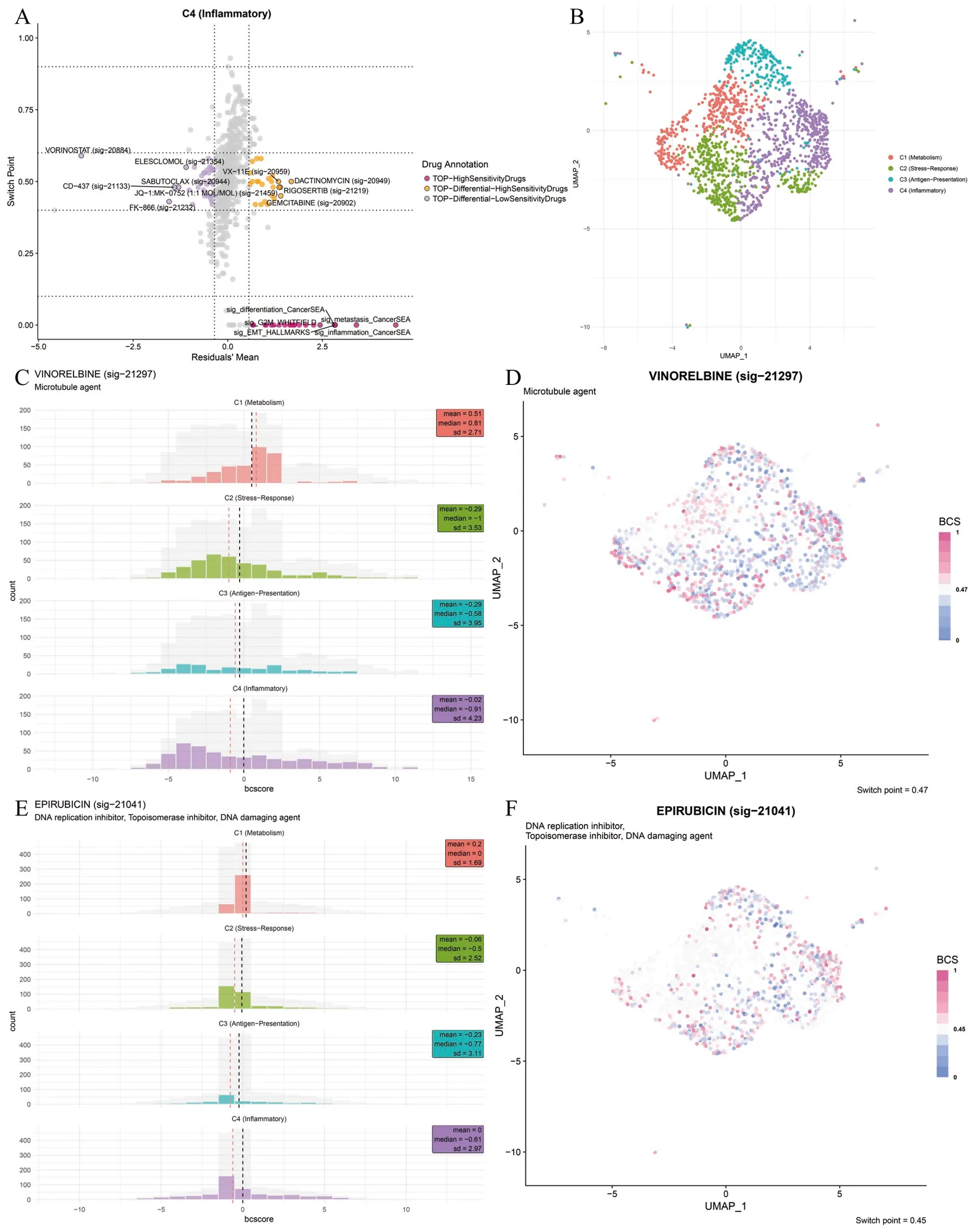
Beyondcell-based predicted drug-response analysis of mast cell subpopulations. **(A)** Four-quadrant plot showing compounds associated with relatively higher or lower predicted sensitivity in the C4 (Inflammatory) subpopulation, ranked by switch point and mean residual. **(B)** UMAP plot of mast cells colored by the four subpopulations (C1–C4). **(C)** Histograms of the BCS distribution for vinorelbine across the four mast cell subpopulations. In each panel, the black dashed line indicates the mean BCS and the red dashed line indicates the median BCS of the corresponding subpopulation. **(D)** UMAP projection of the per-cell BCS for vinorelbine. **(E)** Histograms of the BCS distribution for epirubicin across the four mast cell subpopulations, as described in **(C)**. **(F)** UMAP projection of the per-cell BCS for epirubicin.

### 3.5 The C4 (Inflammatory) mast cell subpopulation correlates with tumor weight and volume

To validate the existence of the C4 (Inflammatory) subpopulation, we established a TNBC model in BALB/c mice using 4T1 cells (**Figure 6A**). We then used flow cytometry to determine whether an ICAM-1-high mast-cell population was present in the murine tumors. Specifically, doublets were excluded based on FSC-A/FSC-H gating, followed by identification of CD45^+^ immune cells. Mast cells were subsequently defined as FcϵRIα^+^CD117^+^ cells, and ICAM-1-high mast cells were further identified within the mast-cell population based on ICAM-1 expression. Flow cytometric analysis of the resulting tumors confirmed the presence of ICAM-1-high mast cells corresponding to the C4 subpopulation in the murine tumors (**Figure 6B**). To investigate whether the proportion of this subpopulation affects tumor growth, we performed a literature search and found that IL-33 can induce an inflammatory state; we therefore administered IL-33 to mice via intraperitoneal injection to examine its effect on the proportion of C4 mast cells and on tumor growth (57–59). The injection dose and route were based on previous studies, and the detailed procedure is described in the Methods and illustrated in the schematic (**Figure 6C**) (60, 61). The mice were euthanized at the third week, and the tumors were harvested (**Figure 6D**). Analysis of the proportion of C4 mast cells in the tumors of the two groups showed that the proportion of C4 was increased in the tumors of IL-33–treated mice (**Figure 6E**). In addition, both the weight and the volume of the tumors in the IL-33 group were greater than those in the control group (**Figures 6F, G**). Correlation analysis further revealed that the proportion of C4 mast cells was positively correlated with both tumor volume and tumor weight (**Figures 6H, I**).

**Figure 6:**
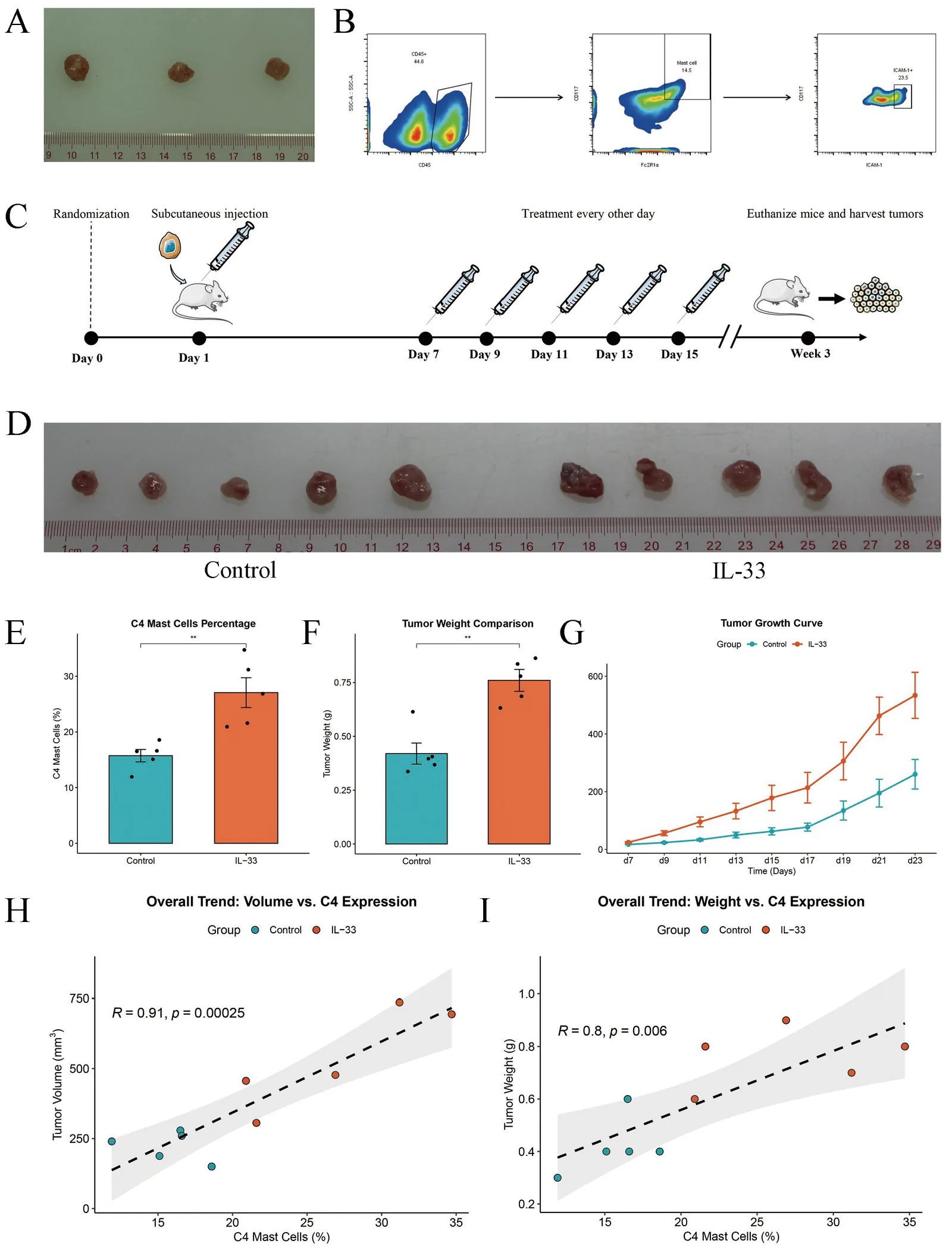
IL-33 treatment expands the C4 (Inflammatory) mast cell subpopulation and promotes tumor growth *in vivo*. **(A)** Representative image of 4T1 subcutaneous tumors harvested from tumor-bearing mice. **(B)** Gating strategy for the flow cytometric identification of the C4 (Inflammatory) mast cell subpopulation: immune cells were gated as CD45^+^, mast cells were identified as CD117^+^ and FcϵR1α^+^, and the C4 subpopulation was defined as ICAM-1^+^ mast cells. **(C)** Schematic of the *in vivo* experimental design. Mice were randomized and subcutaneously injected with 4T1 cells, treated with IL-33 or vehicle every other day from day 7 to day 15, and euthanized at week 3 for tumor harvest. **(D)** Representative images of tumors from the control group (left) and the IL-33–treated group (right). **(E)** Comparison of the proportion of C4 (Inflammatory) mast cells between the control and IL-33–treated groups. Data are presented as the mean ± SEM; Student’s t-test (**p < 0.01). **(F)** Comparison of tumor weight between the control and IL-33–treated groups. Data are presented as the mean ± SEM; Student’s t-test (**p < 0.01). **(G)** Tumor growth curves of the control and IL-33–treated groups, with tumor volume measured every two days. Data are presented as the mean ± SEM. **(H)** Correlation between tumor volume and the proportion of C4 (Inflammatory) mast cells (Pearson’s correlation, R = 0.91, p = 0.00025). **(I)** Correlation between tumor weight and the proportion of C4 (Inflammatory) mast cells (Pearson’s correlation, R = 0.8, p = 0.006).

## 4 Discussion

TNBC, a distinct subtype of breast cancer, has long been associated with poor therapeutic outcomes. With the rise of immunotherapy, research into treatment strategies for this subtype has begun to focus on the TME (9, 17, 62, 63). As a key component of the TME, mast cells have attracted increasing attention; however, their heterogeneity and functional roles within tumors remain incompletely understood. The relationship between mast cells and tumors remains contradictory: mast cells have been found to be associated with poor prognosis and aggressive disease in pancreatic cancer, hepatocellular carcinoma, and cholangiocarcinoma (64–66), whereas in colorectal cancer and HR-positive breast cancer, mast cell infiltration has been reported as a potentially favorable independent prognostic factor (67, 68). This contradiction may be attributable to distinct mast cell subpopulations. In a previous study, Wu et al. identified an antigen-presenting mast cell (apMC) subset associated with the benefit of anti–PD-1 therapy in TNBC (18), whereas in our study, we identified an inflammation-associated mast cell subpopulation (C4) characterized by its involvement in the NF-κB pathway, which may be associated with tumor progression and an unfavorable treatment response.

Through enrichment analysis and transcription factor analysis, we found that the C4 subpopulation was highly enriched in the NF-κB pathway, which has been reported to be associated with tumor promotion and progression. As a central link between chronic inflammation and malignancy, NF-κB connects intrinsic oncogenic signals with extrinsic inflammatory signals and TME remodeling (69, 70). Specifically, during tumorigenesis, NF-κB exacerbates genomic instability through reactive oxygen species (ROS), reactive nitrogen intermediates (RNI), and mutagenic enzymes, while promoting cell survival via anti-apoptotic proteins such as the Bcl family. NF-κB also plays a critical role in driving cancer cell proliferation by promoting cell cycle progression, enhancing proliferation through cyclin D1 and MYC, and sustaining uncontrolled cell division (71, 72). Given that the C4 mast cell subpopulation is highly associated with inflammation and the NF-κB pathway, it may act as a key mediator in NF-κB–driven tumorigenesis, and targeting this subpopulation may help attenuate the oncogenic effects mediated by NF-κB.

In the pseudotime analysis and drug sensitivity analysis, we found that the C4 subpopulation may be associated with treatment response and drug resistance. Given that C4 is highly enriched in the NF-κB pathway, we hypothesize that this subpopulation may contribute to drug resistance and other adverse outcomes through activation of NF-κB signaling. This hypothesis is supported by extensive evidence linking NF-κB to therapeutic resistance across cancers: in breast cancer, NF-κB–driven overexpression of Bcl-2 enhances chemoresistance and metastatic potential (73); in glioblastoma, NF-κB activation has been associated with temozolomide resistance (74); and in human gastric cancer, NF-κB has likewise been found to promote chemoresistance (75). Collectively, these findings raise the possibility that targeting the C4 subpopulation may help attenuate NF-κB–associated resistance and improve therapeutic outcomes, a possibility that warrants further investigation.

Another prominent feature of the C4 mast cell subpopulation is its important role in processes such as cell adhesion, motility, and EMT. As a signature gene of this subpopulation, ICAM-1 has been reported to activate the EMT program through the TGF-β/SMAD signaling pathway in TNBC (76). In bladder cancer, ICAM-1 can regulate the EMT process through the N-cadherin/SRC/AKT/GSK-3β/β-catenin signaling axis (77). Moreover, VCAM-1, which was closely associated with the C4 mast cell subpopulation in the cell–cell communication analysis, has also been reported to be related to EMT (78). In addition, the genes perturbed upon ICAM-1 knockout likewise play important roles in EMT. For instance, AHNAK may promote cell proliferation and migration in pancreatic ductal adenocarcinoma (PDAC), thereby influencing PDAC prognosis through EMT (29); MYH9 regulates the EMT process of glioma cells via β-catenin (30); and overexpression of FMNL3 induces EMT in nasopharyngeal carcinoma (31). ZEB2, in turn, may promote metastatic dissemination in melanoma (28). These observations are highly consistent with the functions of NF-κB, which has been reported to promote tumor cell–endothelial interactions—critical for extravasation—through the cell adhesion molecules it mediates, such as VCAM-1, ELAM-1, and ICAM-1 (48). Furthermore, NF-κB enhances metastatic potential and chemoresistance by upregulating the expression of transcription factors such as ZEB2 to promote EMT (79).

In the cell–cell communication analysis, we also found that the C4 mast cell subpopulation was closely associated with CD8^+^ T cells, to which it sent outgoing signals through CD274 (PD-L1)–PDCD1 (PD-1), LGALS9 (galectin-9)–HAVCR2 (TIM-3), Nectin-2–TIGIT, FASL–FAS, and MIF–(CD74+CXCR4). A prominent feature of these signals is their potential involvement in immune suppression and T-cell exhaustion. As an important target of immunotherapy, the PD-1/PD-L1 axis has been found to attenuate host antitumor immune responses and promote tumor immune evasion in various malignancies (43). When TIM-3 binds to galectin-9, it can downregulate IFN-γ production and T-cell proliferation; in an *in vitro* experiment, the addition of galectin-9 induced the death of TH1 cells in a TIM-3–dependent manner (37, 44). Nectin-2, through its binding to TIGIT, can negatively regulate T-cell activation (45). The Fas/FasL pathway maintains immune homeostasis by killing activated T cells (46). MIF–CD74/CXCR4 signaling may lead to the aberrant activation of CD8^+^ T cells, thereby accelerating their progression toward an exhausted state (80). As the principal cytotoxic cells in the tumor microenvironment, CD8^+^ T cells play a pivotal role in immunotherapy. Although these inhibitory relationships were inferred from communication analysis and require further experimental validation, they suggest that the potential suppression of CD8^+^ T cells by C4 mast cells may partly explain the association of this subpopulation with treatment resistance.

Previous studies have shown that exposure to IL-33 induces the production of pro-inflammatory mediators and the activation of NF-κB in mast cells, a state that closely resembles that of the C4 subpopulation identified in our study (57–59). We therefore sought to examine whether the changes in mast cell behavior following IL-33 exposure correspond to alterations in specific mast cell subpopulations. After IL-33 administration, we found that the proportion of the C4 subpopulation in the tumors was increased, accompanied by an increase in tumor size and weight. These findings further support our conclusion that C4 represents a pro-tumor subpopulation.

This study has several limitations. First, a distinct neutrophil population was not identified in the scRNA-seq dataset used in this study. Because neutrophils have relatively low RNA content and are particularly susceptible to loss during tissue dissociation and single-cell sample preparation, their abundance may be underestimated in conventional scRNA-seq datasets. Consequently, the present analysis may not fully capture the myeloid-cell composition of the TNBC tumor microenvironment. Although this limitation does not directly affect our primary analysis of mast-cell heterogeneity, future studies incorporating neutrophils and other potentially underrepresented myeloid populations will be important for achieving a more comprehensive understanding of the TNBC immune microenvironment and its intercellular interactions. In addition, although the scRNA-seq analysis in this study was based on clinical tumor samples from patients with TNBC, the experimental validation was primarily performed in a murine model. Independent validation of the C4 mast-cell population by IHC or IF in human TNBC tissues is currently lacking. Future studies incorporating histological and spatial validation in independent clinical cohorts will be important to further confirm the existence and clinical relevance of this population.

## 5 Conclusion

In this study, we identified an inflammation-associated mast cell subpopulation (C4) in TNBC that is characterized by its involvement in the NF-κB pathway. By integrating single-cell analysis with experimental validation, we demonstrated that C4 is associated with tumor progression, an unfavorable treatment response, and the suppression of antitumor immunity, and that its expansion promotes tumor growth *in vivo*. These findings identify C4 as a potential contributor to TNBC progression and a promising target for future therapeutic exploration. A deeper understanding of this subpopulation may shed light on the mechanistic link between chronic inflammation and tumor progression.

## Data Availability

The original contributions presented in the study are included in the article/**Supplementary Material**. Further inquiries can be directed to the corresponding authors.

## Supplementary material

Supplementary Figure 1 Virtual knockout analysis of ICAM1, global communication patterns, and mast-cell subset distribution across treatment regimens in the TNBC tumor microenvironment. **(A)** Bar plot showing the top 20 genes most strongly affected by the in silico knockout of ICAM1, ranked by manifold distance (fold change). **(B)** Volcano plot of the differential regulation induced by ICAM1 knockout. The horizontal dashed line denotes the significance threshold, and the vertical dashed lines denote Z-score cut-offs of ±2; significantly altered genes are highlighted in red. **(C)** Heatmap of the incoming communication patterns, showing the association between the identified patterns and the signaling pathways. **(D)** Heatmap of the outgoing communication patterns, showing the association between the identified patterns and the signaling pathways. **(E)** Box plots showing the relative proportions of the four mast-cell subsets (C1–C4) across patients receiving different treatment regimens.

Supplementary Figure 2 BCS distributions of representative breast cancer–related agents across mast cell subpopulations. **(A)** Histograms of the BCS distribution for paclitaxel across the four mast cell subpopulations (C1–C4). In each panel, the black dashed line indicates the mean BCS and the red dashed line indicates the median BCS of the corresponding subpopulation. **(B)** UMAP projection of the per-cell BCS for paclitaxel. **(C)** Histograms of the BCS distribution for gemcitabine across the four mast cell subpopulations, as described in **(A)**. **(D)** UMAP projection of the per-cell BCS for gemcitabine. **(E)** Histograms of the BCS distribution for olaparib across the four mast cell subpopulations, as described in **(A)**. **(F)** UMAP projection of the per-cell BCS for olaparib. **(G)** Histograms of the BCS distribution for alpelisib across the four mast cell subpopulations, as described in **(A)**. **(H)** UMAP projection of the per-cell BCS for alpelisib.
